# Associations Between Prayer Practices, Healthy Behaviors, and Depression Among Iranian Medical Students: A Cross‐Sectional Structural Equation Modeling Study

**DOI:** 10.1002/hsr2.73243

**Published:** 2026-09-15

**Authors:** Mehrdad Karimi, MoradAli Zareipour, Esmayil Zeynali, Rahim Sharafkhani

**Affiliations:** ^1^ Department of Public Health Khoy University of Medical Sciences Khoy Iran; ^2^ Department of Medicine Khoy University of Medical Sciences Khoy Iran

**Keywords:** cross‐sectional studies, depression, health behavior, prayer practices, religion, structural equation modeling, students

## Abstract

**Background:**

Prayer is a concept that indicates the level of an individual's commitment, attachment, and adherence to worship in life. However, evidence regarding its associations with health behaviors and depression remains limited. This study aimed to examine the associations between prayer practices, healthy behaviors, and depression among students of the University of Medical Sciences.

**Methods:**

This study employed a cross‐sectional design. A total of 362 students with a mean age of 21.67 ± 2.98 years participated in the study. Data were collected using questionnaires on prayer, health behaviors, and depression. The data were analyzed using IBM SPSS AMOS and IBM SPSS Statistics version 24. Structural equation modeling was used to evaluate the interrelationships between research variables and to test the conceptual model of the study.

**Results:**

The results of this study showed that prayer had a significant direct relationship with a reduction in smoking (β = 0.27, *p* < 0.001) and a significant indirect relationship with a reduction in depression scores (β = −0.13, *p* = 0.03) among students. The relationship of prayer with a healthy diet and physical activity was not significant (*p* > 0.05).

**Conclusion:**

Given the cross‐sectional design, causal relationships cannot be inferred. Prayer practices were associated with reduced smoking behavior and lower depression scores among medical students; however, these findings should be interpreted cautiously due to methodological limitations, including self‐reported data and the non‐experimental design. Longitudinal studies are recommended to clarify causal pathways.

## 1 Introduction

Prayer is a universal and integral component of religious practice, involving communication with a transcendent being or power. It serves various purposes, such as expressing gratitude, seeking guidance, requesting assistance, confessing sins, or praising God [1, 2]. In Islamic contexts, prayer (salat) represents a structured and obligatory religious practice performed five times daily as one of the Five Pillars of Islam [2]. salat can be performed individually or collectively, privately or publicly, verbally and with or without specific rituals. It is a central aspect of many religions, particularly Islam, which mandates five daily salat as one of the five pillars of faith [3]. For conceptual clarity, it is important to distinguish between religiosity, spirituality, and prayer practices. Religiosity refers to organized religious beliefs and behaviors, spirituality reflects personal experiences of meaning and transcendence, whereas prayer practices represent a specific behavioral dimension of religious commitment that can be operationally measured [4, 5].

The concept of prayer reflects the degree of commitment, attachment, and adherence to prayer in an individual's life [6]. prayer is influenced by various factors, including personal beliefs, religious teachings, social norms, and psychological needs, and it can vary across different situations, contexts, and stages of life [7]. prayer encompasses behavioral, cognitive, emotional and spiritual dimensions, profoundly impacting one's well‐being and quality of life [6].

Previous research has explored the relationship between prayer and various health aspects, such as physical, mental, and social health [8]. Some studies have reported positive associations between prayer and health outcomes, including lower blood pressure, reduced stress, enhanced coping, increased happiness, and improved social support [2]. Conversely, other studies have found negative or mixed associations, such as higher anxiety, depression, guilt, and religious struggle [9]. These inconsistent findings suggest that the relationship between prayer and health is complex and multifaceted, potentially moderated and mediated by factors such as the type, frequency, quality, and motivation of prayer, as well as the individual and contextual characteristics [10]. One health domain that has garnered considerable attention concerning prayer is healthy behaviors, defined as “actions undertaken by individuals to maintain, enhance, or restoring health”. These behaviors include physical exercise, a balanced diet, adequate sleep, smoking cessation, alcohol moderation, and preventive care [11]. Healthy behaviors are critical determinants of health status and disease prevention and can influence the course and outcomes of chronic conditions, such as diabetes, cardiovascular disease, and cancer [12].

From a theoretical perspective, healthy behaviors may serve as a mediating pathway linking prayer practices to depression. Prayer may enhance self‐regulation, stress reduction, and social connectedness, which in turn may promote healthier lifestyle behaviors and contribute to improved mental health outcomes [13, 14] Another frequently studied health domain in relation to prayer is depression, a common and serious mental disorder characterized by persistent feelings of sadness, hopelessness, and loss of interest or pleasure in daily activities [15]. Depression impairs various aspects of functioning, including cognition, emotion, motivation, and behavior, and increase the risk of morbidity and mortality from physical illnesses such as cardiovascular disease, diabetes, and stroke [16, 17]. Depression is a significant public health problem, affecting over 264 million people worldwide and imposing substantial social and economic burdens [18].

The relationship between prayer and healthy behaviors and depression has been investigated primarily among Muslim populations, given the centrality of prayer in Islam. Some studies have found positive associations between prayer and healthy behaviors, such as physical activity, a healthy diet, and smoking cessation, and negative associations with depression, suggesting that prayer may have protective and beneficial health effects [19, 20]. However, previous studies have largely focused on bivariate or descriptive associations, and limited research has simultaneously tested the interrelationships among prayer practices, multiple health behaviors, and depression using advanced analytical techniques such as structural equation modeling [21, 22]. Thus, a clear research gap exists regarding the mediating role of healthy behaviors within this context.

To better examine these complex relationships and potential indirect effects, advanced statistical approaches such as structural equation modeling (SEM) are recommended. SEM allows simultaneous testing of direct and indirect relationships among observed and latent variables and is appropriate for evaluating mediation models in behavioral research [23, 24, 25, 26].

Prayer and its relationship to healthy behaviors and depression among medical students requires careful attention, because medical students are at higher risk of developing depression due to academic pressures and stress. Examining these relationships may help inform strategies to improve students' mental health and well‐being. Accordingly, the present study aimed to investigate the relationships between prayer practices, healthy behaviors, and depression among Iranian medical students using a cross‐sectional structural equation modeling approach.

To address methodological clarity, the study formulated explicit, testable hypotheses distinguishing direct and indirect pathways: H1: Prayer practices are directly associated with selected healthy behaviors (physical activity, healthy diet, and non‐smoking behavior). H2: Prayer practices are directly associated with lower levels of depression. H3: Selected healthy behaviors are directly associated with lower levels of depression. H4: Prayer practices are indirectly associated with depression through selected healthy behaviors (mediation effect).

## 2 Methods

The present study is cross‐sectional. Given the cross‐sectional and observational nature of the design, the identified relationships reflect associations rather than causal effects, and temporal ordering among variables cannot be definitively established. The research population comprised all students at the Khoy Medical Sciences Faculty (500 individuals), examined through a census sampling method. A total of 362 students agreed to participate in the study. (response rate = 72.4%). Although a census approach was intended, participation was voluntary; therefore, the possibility of self‐selection and volunteer bias should be acknowledged. Ethical approval was obtained (IR.KHOY.REC.1402.006), and the researchers explained the objectives of the research to the students and secured their informed consent.

After providing an explanation of the study's goals and the process for completing the questionnaire, and assuring the confidentiality and anonymity of the information gathered, the researchers requested the participating students to read and respond to the questions in a self‐reporting manner. The students, categorized by field of study and academic entry year, completed the questionnaires over 15 days, either before or after class. The questionnaire included four sections: demographic information, adherence to prayer, health behaviors, and depression severity.

### 2.1 Inclusion and Exclusion Criteria

Inclusion criteria for the study were being a student in any field at the Khoy Medical Sciences Faculty and providing informed consent to participate in the study. Participation was voluntary. Students who did not complete more than 10% of the questionnaire were excluded from the study to ensure data quality and analytical accuracy. Additionally, students with documented psychiatric disorders or those who had active records in the university counseling center were excluded from the study. This criterion was applied to minimize potential confounding effects of existing mental health conditions on depression scores and related variables.

For the remaining missing data, Full Information Maximum Likelihood (FIML) estimation was applied within the structural equation modeling framework to reduce bias associated with incomplete responses.

Study Period: The present cross‐sectional study was carried out over a period from September 18, 2022, to December 7, 2024.

### 2.2 Sample Size and Generalizability

No a priori power analysis was conducted prior to data collection. However, the final sample size (*N* = 362) exceeds commonly recommended minimum thresholds for SEM analyses (e.g., *N* ≥ 200 or a ratio of 10–20 participants per estimated parameter), suggesting adequate statistical power for detecting moderate effects. The absence of a pre‐study power calculation is acknowledged as a methodological limitation. Because the sample was drawn from a single medical sciences faculty, the generalizability of the findings to students from other universities or broader populations may be limited. Replication in multi‐center and more diverse samples is recommended to strengthen external validity.

## 3 Measurement Tools

### 3.1 Prayer Questionnaire

The prayer Questionnaire was developed by Anisi et al. (2010) to assess the significance and role of prayer in individuals' lives. This 50‐question survey evaluates various aspects of prayer, including its effectiveness in personal and social contexts, adherence to recommended practices, commitment and earnestness in performing prayers, and mindfulness and presence of heart during prayer [27]. This questionnaire is a standardized instrument that has previously been psychometrically validated and widely used in Iranian populations. Therefore, based on its established factorial structure in prior studies, conducting a new confirmatory factor analysis (CFA) was not required; however, additional reliability and validity assessments were performed in the present study.

The prayer scale uses a Likert scale for responses, with options ranging from “never” to “always” and “strongly disagree” to “strongly agree”. Scores are assigned as follows:

- 0 for “never” or “strongly disagree”
- 1 for “very rarely” or “disagree”
- 2 for “sometimes” or “somewhat agree”
- 3 for “often” or “agree”
- 4 for “always” or “strongly agree”

The total score ranges from 0 to 200, with higher scores indicating greater emphasis on prayer. The prayer scale has undergone rigorous psychometric validation, demonstrating both credibility and validity. It effectively measures individuals' attitudes and practical commitment to prayer.

The validity of the prayer has been confirmed through various methods, including content validity, concurrent validity, and construct validity. Its reliability has been established through internal consistency (Cronbach's alpha) and stability (test‐retest reliability). The reliability of this tool has been reported at 92% through internal consistency (Cronbach's alpha calculation), while the test‐retest reliability over an interval of 1 week is 96%.

In the present study, internal consistency (Cronbach's alpha), composite reliability (CR), and average variance extracted (AVE) were calculated to further examine reliability and convergent validity within the current sample.

### 3.2 Health Behavior Questionnaire

To assess health behaviors, a standardized questionnaire developed by Ahmadi et al. (2019) was utilized [28]. The Health Behavior Questionnaire includes items on physical activity, diet, and smoking of cigarettes and hookah, with responses in a “yes” or “no” format. This questionnaire has been used in various studies in Iran by Ahmadi and colleagues (2019). Given that this instrument has an established psychometric background in Iran, its structural validity had been previously confirmed. Nevertheless, in the present study, reliability and validity indices (Cronbach's alpha, CR, and AVE) were re‐evaluated.

The validity of the Health Behavior Questionnaire was established through content validity, and its reliability was determined by a Cronbach's alpha coefficient of 0.70 [28]. It should be noted that physical activity was measured using a single indicator, which represents a methodological limitation in SEM. Additionally, the dichotomous (yes/no) response format may reduce variability and sensitivity of measurement. Subscales with AVE values below the conventional threshold (0.50) were interpreted cautiously as part of convergent validity assessment.

### 3.3 Patient Health Questionnaire (PHQ‐9)

The Patient Health Questionnaire (PHQ‐9) is a multipurpose tool for screening, diagnosing, monitoring, and measuring the severity of depression. It was psychometrically validated by Dadfar et al. [29]. The PHQ‐9 integrates the DSM‐IV diagnostic criteria for depression and other major depressive symptoms into a brief self‐report instrument. In this study, the PHQ‐9 was used as a screening instrument to assess depressive symptom severity rather than as a diagnostic tool. Therefore, it does not replace clinical psychiatric assessment. The questionnaire consists of 9 questions, with responses on a Likert scale ranging from “never” to “almost every day.” Scores are assigned as follows:

- 0 for “never”
- 1 for “several days”
- 2 for “more than half the days”
- 3 for “almost every day”

The total score ranges from 0 to 27, with higher scores indicating more significant signs of depression in an individual. Information regarding participants' prior psychiatric diagnoses or use of psychotropic medications was not collected, which is acknowledged as a limitation in interpreting depression scores.

### 3.4 Statistical Methods

In the current study, descriptive statistics, including the mean ± standard deviation and frequency (percentage) were used to summarize the data. The Pearson correlation coefficient was used to assess the linear relationships among the research variables. To evaluate the relationships between commitment to prayer, health‐oriented behaviors (such as a healthy diet, physical activity, and smoking), and depression among students, Structural Equations Modeling (SEM) was utilized. Given the cross‐sectional and observational nature of the study design, SEM was employed to examine associative relationships among variables rather than to establish causal effects. Therefore, the estimated structural paths should be interpreted as correlational associations, and no definitive causal inferences can be drawn.

**Figure 1:**
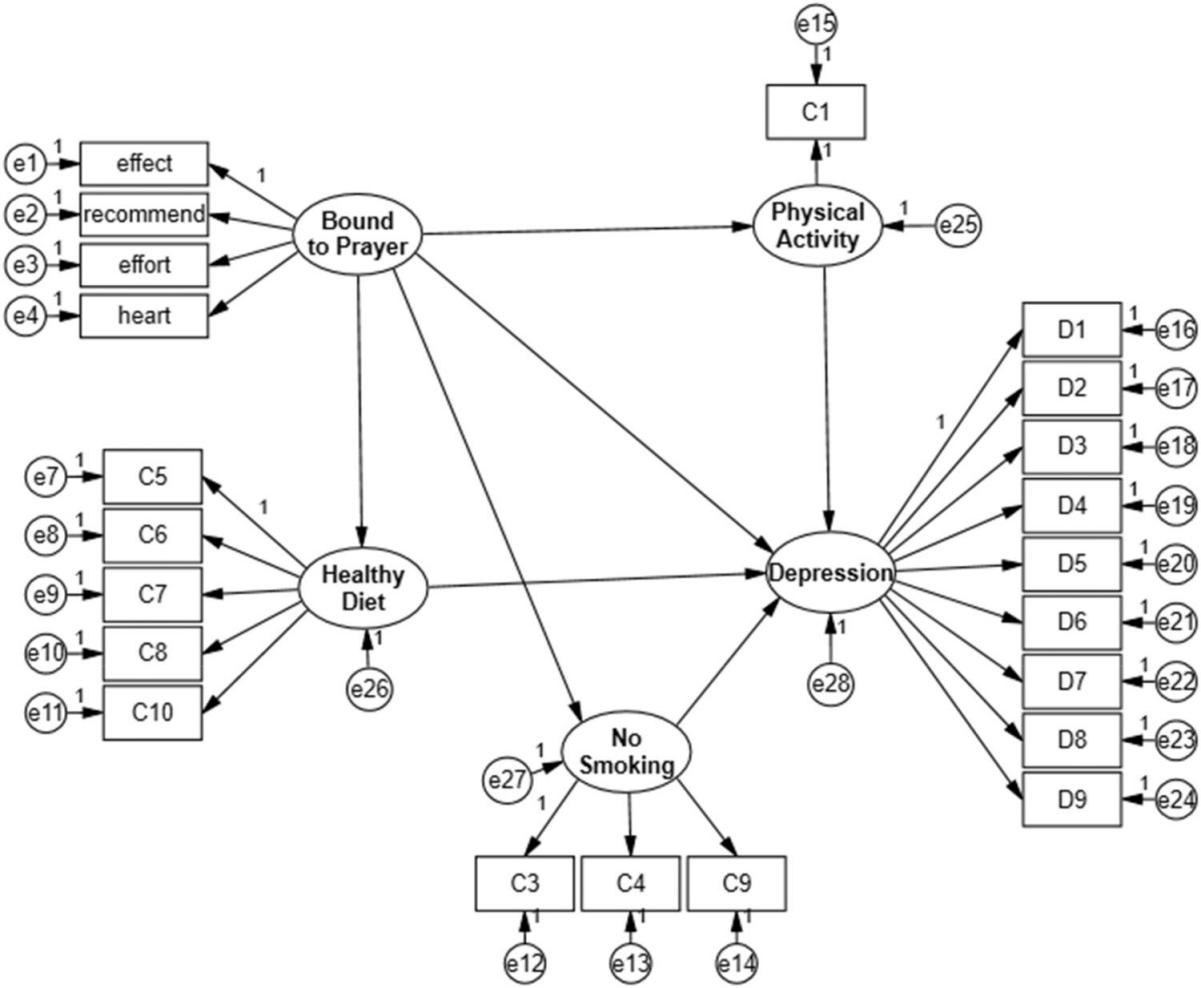
Conceptual framework of the interrelationship among prayer, health behaviors, and depression.

In this structural model, the latent variable representing commitment to prayer was treated as the exogenous independent variable. Health behaviors, comprising three latent variables physical activity, healthy diet, and abstinence from smoking and hookah served as mediators. The latent variable of depression was considered the ultimate dependent variable (Figure 1). Although directional paths were specified based on theoretical assumptions, the temporal ordering of variables cannot be empirically confirmed within a cross‐sectional framework. Accordingly, mediation effects are interpreted as statistical indirect associations rather than evidence of true temporal mediation.

The validity and reliability of the research constructs were evaluated using Cronbach's alpha, composite reliability coefficient (CR), and the average variance extracted (AVE). The latent construct of commitment to prayer was measured with four indicators effect, recommend, effort, heart. Physical activity was measured with one indicator, healthy nutrition with five indicators, non‐consumption of cigarettes and hookah with three indicators, and depression with nine indicators present in the conceptual model.

Model fit indices tested included Chi‐square, degrees of freedom (DF^1^), Chi‐square to DF ratio, Standardized Root Mean Square Residual (SRMR), Root Mean Square Error of Approximation (RMSEA), Goodness of Fit Index (GFI), Adjusted Goodness of Fit Index (AGFI), Normed Fit Index (NFI), Incremental Fit Index (IFI), Comparative Fit Index (CFI), and Tucker‐Lewis Index (TLI). These indices were calculated for the structural model, and the results and acceptable ranges for model fit were discussed. In SEM, acceptable fit thresholds for various indices are as follows:

- The Chi‐square to degrees of freedom ratio should be 5 or less for a good model fit.
- RMSEA values should be 0.06 or less for a good fit, with values up to 0.08 being acceptable in some cases.
- SRMR should be less than 0.08 for a good fit.
- GFI and AGFI values should be 0.90 or higher for an acceptable fit.
- NFI, CFI, and TLI values should also be above 0.90 for a model to be considered well‐fitting [30, 31].

These thresholds may vary slightly depending on the model's complexity and the specific research context. It is important to consider the overall pattern of fit indices rather than relying on a single measure. Model modification procedures were conducted cautiously and were not based solely on statistical fit indices. Modifications (adding or removing paths) were performed only when two criteria were met simultaneously [1]: a substantial modification index (MI > 15), and [2] clear theoretical justification based on established literature. Modification indices were considered in conjunction with theoretical plausibility rather than as independent criteria for model revision. This approach was used to minimize the risk of overfitting and data‐driven model specification.

Subsequently, the modified structural model tested the relationships between research variables, considering student gender, semester, household economic status, and student residence. Weak relationships with a significance level of *p* > 0.20 were removed to avoid clutter and complexity in interpretation. However, non‐significant direct paths that were part of the hypothesized mediation structure (e.g., prayer to physical activity and prayer to healthy diet) were retained in the model to avoid biased indirect effect estimation, consistent with standard SEM practices. Ultimately, based on an acceptable reduction in the Chi‐square value, some theoretically justifiable relationships that improved model fit were added. The final revised model served as the basis for evaluating the relationships among research variables.

Structural equation analyses were conducted using AMOS 24, while descriptive indices and routine analyses were performed using SPSS 24.

## 4 Results

### 4.1 Study Population

Table 1 provides demographic information on 362 college students. The mean age of the students was 21.67 ± 2.93 years old. The majority of the students were female (55.5%), unmarried (88.7%), and studying nursing (33.4%). The students were also evenly distributed between dormitory (71.0%) and native (29.0%) residency. The students' economic status was also evenly distributed between weak (14.9%), middle (66.3%), and good (18.8%). The median semester of students was 3.0, and the average score of the semester past in the students studied was 16.81 ± 1.65.

**Table 1:** Base characteristics of the study population.

Variables	*n* (%), mean ± SD
Age	21.67 ± 2.93
Sex	
Male	161 (44.5)
Female	201 (55.5)
Marital status	
Single	321 (88.7)
Married	41 (11.3)
Field of study	
Environmental health	49 (13.5)
Public health	49 (13.5)
Nursing	121 (33.4)
Surgical technologist	47 (13.0)
MD	49 (13.5)
EMS	47 (13.0)
Residency	
Dormitory	257 (71.0)
Native	105 (29.0)
Economic status	
Weak	54 (14.9)
Middle	240 (66.3)
Good	68 (18.8)
Semester	3.0 (1.0–7.0)*
Score	16.81 ± 1.65

*Median (Q1–Q3).

### 4.2 Interpretation of Validity and Reliability Indices

Table 2 shows the mean and standard deviation of the main variables in the study along with reliability and validity indices including Cronbach's alpha, composite reliability (CR), and average variance extracted (AVE).

**Table 2:** Mean and standard deviation of study variables along with their validity and reliability assessment.

Variables	No of items	Mean ± SD	Cronbach's alpha	CR	AVE
Prayer	49	118.93 ± 45.18	0.98	0.91	0.55
Effect	14	38.93 ± 14.13	0.96	0.95	0.66
Recommend	12	24.66 ± 10.96	0.91	0.89	0.50
Effort	13	28.48 ± 12.68	0.95	0.95	0.64
Heart	10	26.86 ± 9.85	0.93	0.95	0.74
Heath behavior	9	18.23 ± 4.75	0.74	0.73	0.40
Physical	1	1.70 ± 0.95	—	—	—
Diet	5	9.26 ± 3.42	0.78	0.90	0.53
No smoke	3	7.26 ± 2.07	0.65	0.93	0.43
PHQ	9	6.93 ± 5.29	0.88	0.90	0.52

The Cronbach's alpha coefficient is a measure of internal consistency, which indicates how well the items in a scale measure the same construct. In general, a Cronbach's alpha of 0.70 or higher is considered acceptable. The composite reliability coefficient (CR) is a more conservative measure of internal consistency than Cronbach's alpha. It is calculated by dividing the average variance extracted (AVE) by the number of items in the scale. In general, a CR of 0.70 or higher is considered acceptable. The average variance extracted (AVE) is a measure of how much variance in the construct is explained by the scale. In general, an AVE of 0.50 or higher is considered acceptable. The results of the validity and reliability analyses show that all of the scales have acceptable levels of internal consistency. The Cronbach's alpha coefficients for all of the scales are above 0.70, and the CR coefficients are above 0.70. Except for the health behavior scale, the AVE coefficients are also above 0.50 for all of the scales.

Discriminant validity was assessed using the Fornell‐Larcker criterion [32]. According to this criterion, discriminant validity is established when the square root of the Average Variance Extracted (AVE) for each construct is greater than its highest correlation with any other construct in the model. As shown in Table 2, the square root of AVE for prayer (√0.55 = 0.74) and for depression (√0.52 = 0.72) were both substantially higher than their inter‐construct correlation (r = −0.16, from Table 3). These results confirm that prayer and depression are empirically distinct constructs, and the discriminant validity of the measurement model is supported.

**Table 3:** Pearson correlation test among prayer, health behaviors, and depression.

Variables	1	2	3	4	5	6	7	8	9
1. Prayer									
2. Effect									
3. Recommend									
4. Effort									
5. Heart									
6. Health behavior	0.16**	0.21**	0.13*	0.10	0.15**				
7. Physical	0.02	0.01	0.06	−0.01	0.01				
8. Diet	0.08	0.11*	0.09	0.04	0.06				
9. No smoke	0.22**	0.31**	0.13*	0.15**	0.24**				
10. Depression	−0.16**	−0.16**	−0.12*	−0.17**	−0.15**	−0.25**	−0.13**	−0.14**	−0.29**

*p < 0.05

**p < 0.01.

### 4.3 Pearson Correlation Matrix of the Study Variables

Table 3 shows the Pearson correlation matrix for the study variables. The table shows the correlation coefficient between each pair of variables, as well as the p‐value for the correlation. The results of the correlation analysis show that there were significant positive correlations between the health behavior scale and prayer (r = 0.16, *p* < 0.01) as well as its subscales including effect (r = 0.21, *p* < 0.01), recommend (r = 0.13, *p* < 0.05), and heart (r = 0.15, *p* < 0.01). The correlation between health behavior scale and effort subscale was not significant (r = 0.16, *p* > 0.05).

Regarding health behavior subscales, the correlations between no smoke and prayer (r = 0.22, *p* < 0.01) and its subscale such as effect (r = 0.31, *p* < 0.01), recommend (r = 0.13, *p* < 0.05), effort (r = 0.15, *p* < 0.01), and heart (r = 0.24, *p* < 0.01) were positively significant. The correlation between physical activity and prayer and its dimensions was not significant, and the correlation between nutrition and prayer was only significant in one case (effect; r = 0.11, *p* < 0.05).

Finally, the negative correlations between both variables prayer (r = −0.16, *p* < 0.01) and health behavior (r = −0.25, *p* < 0.01) with depression were significant. In this way, healthy behavior and its dimensions, as well as prayer and its dimensions, have significantly reduced depression in students.

## 5 Structural Model Results

### 5.1 Conceptual Model

The model shown in Figure 1 is a structural equation model (SEM) that examines the relationship between three variables prayer, healthy behaviors, and depression. Based on this structural model, prayer is considered as an external independent variable, healthy behaviors including healthy diet smoking cessation, and physical activity are considered as mediating variables, and depression is considered as the endogenous dependent variable. The model hypothesized that prayer is positively correlated with healthy behaviors, and healthy behaviors, in turn, are negatively correlated with depression. The model also hypothesized that healthy behaviors can mediate the relationship between the bond to prayer and depression.

Prayer, healthy behaviors including a healthy diet, physical activity, and smoking cessation are latent variables, which means that they are not directly observable. They are measured by a set of observed variables that are specified in the model. The effect, recommend, effort, and heart are indicators of the prayer construct, while C1 to C10 are indicators of healthy behaviors. Depression is a latent construct measured by a set of survey items that assess the person's symptoms of depression (D1 to D9).

## 6 Modified Structural Model

**Figure 2:**
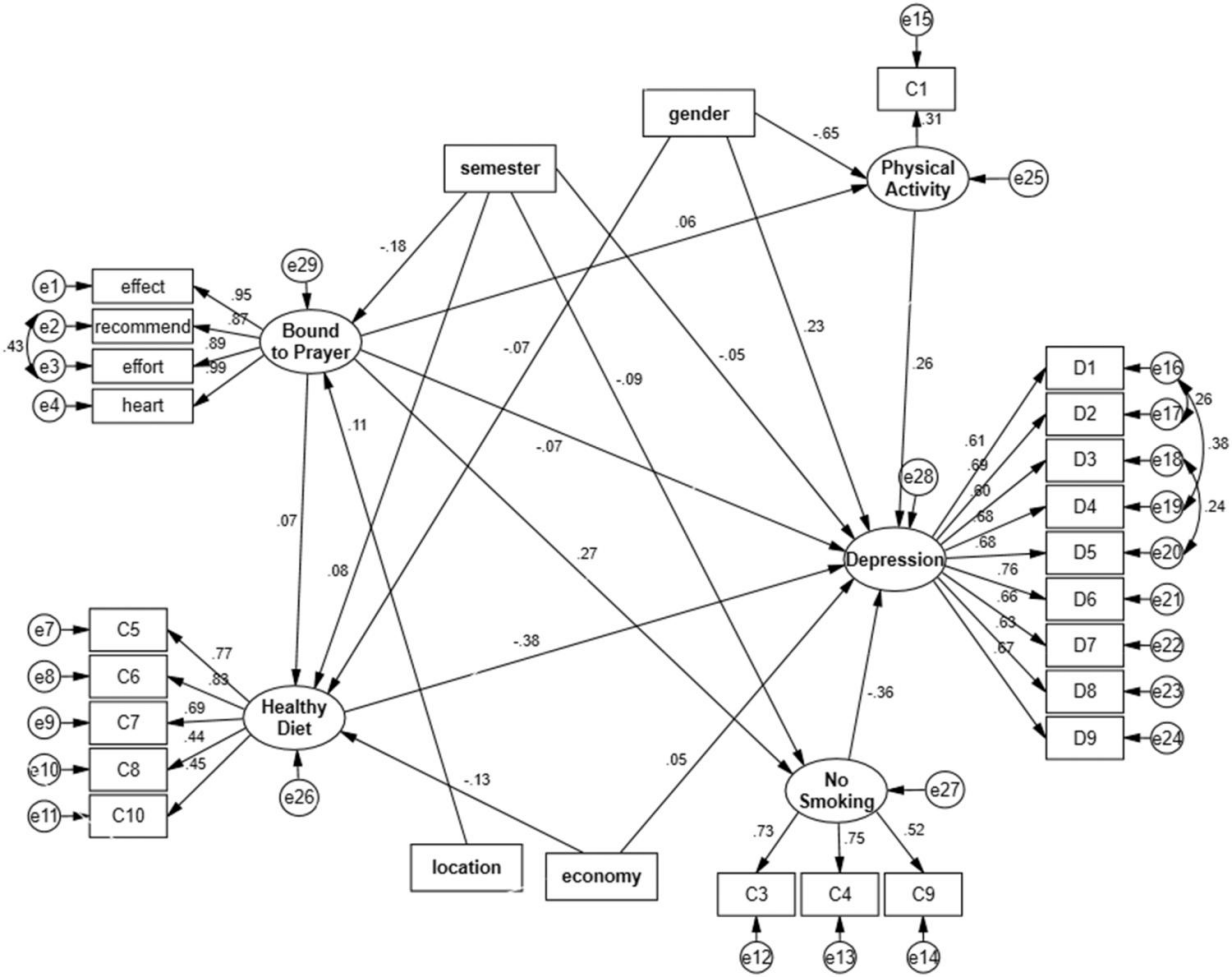
Modified model of the interrelationship among prayer, health behaviors, and depression.

The results of the modified model of the relationship between the study variables, with the moderation effect of important and effective demographic variables in this relationship, are shown in Figure 2. The model fit indices are calculated and reported below the figure, indicating the appropriate and acceptable fit of the tested structural model. In the modified structural equation model, the relationships between demographic variables and the main study variables with a p‐value greater than 0.20 were removed from the model.

Fit indices: Chi‐square = 640.31, DF = 281, Chi‐square to DF = 2.28, SRMR = 0.060, RMSEA = 0.060, GFI = 0.89, AGFI = 0.88, NFI = 0.89, IFI = 0.93, CFI = 0.92, TLI = 0.91.

The results of the test of direct and indirect regression effects based on the modified structural equation model are reported in Table 4. Based on the direct relationships assessed, male students had significantly less physical activity than female students (β = −0.652, *p* < 0.001), and prayer significantly decreased with increasing academic term (β = −0.184, *p* < 0.001). Native students had a higher score on prayer than non‐native or dormitory students (β = 0.111, *p* = 0.03), and the physical activity significantly decreased with increasing economic status from poor to good (β = −0.126, *p* = 0.03). In the evaluation of the direct relationship between prayer and healthy behaviors, prayer showed a significant direct relationship with non‐smoking (β = 0.274, *p* < 0.001), and showed no significant relationship with physical activity and healthy diet. The direct relationship of prayer with depression was not significant (*p* > 0.05). In the test of the direct relationship between healthy behaviors and depression in students, non‐smoking was associated with a decrease in depression (β = −0.363, *p* = 0.03), and the relationship between physical activity and healthy eating with depression was not significant (*p* > 0.05).

**Table 4:** Structural model results: standardized direct and indirect associations among variables based on the modified model.

		Direct effect		Indirect effect		Total effect	
Independent	Dependent	β	*p*‐value	β	*p*‐value	β	*p*‐value
Gender (male)	Prayer						
	Physical act	−0.652	< 0.001				
	Healthy diet	−0.075	0.21				
	Depression	0.229	0.21	−0.142	0.27	0.087	0.12
Semester	Prayer	−0.184	< 0.001				
	Physical act			−0.102	0.64	−0.102	0.64
	Healthy diet	0.076	0.17	−0.013	0.20	0.063	0.26
	No smoking	−0.085	0.16	−0.050	< 0.001	−0.135	0.03
	Depression	−0.047	0.45	0.036	0.58	−0.011	0.85
Residency (dormitory)	Prayer	0.111	0.03				
	Physical act			0.007	0.51	0.007	0.51
	Healthy diet			0.008	0.18	0.008	0.18
	No smoking			0.030	0.02	0.030	0.02
	Depression			−0.020	0.02	−0.020	0.02
Economic	Prayer						
	Physical act						
	Healthy diet	−0.126	0.03				
	No smoking						
	Depression	0.052	0.41	0.048	0.17	0.100	0.09
Prayer	Physical act	0.064	0.70				
	Healthy diet	0.069	0.24				
	No smoking	0.274	< 0.001				
	Depression	−0.073	0.32	−0.131	0.03	−0.204	0.01
Physical act	Depression	0.261	0.37				
Healthy diet	Depression	−0.381	0.21				
No smoking	Depression	−0.363	< 0.001			−0.363	< 0.001

Indirect effect evaluations revealed that the indirect effect of the semester on non‐smoking was significant (β = −0.05, *p* < 0.001), which indicates the mediating role of prayer in the relationship between these two variables. The results also showed that the indirect effect of native‐ness on non‐smoking and depression was significant. This means that native students were more likely to be non‐smokers (β = 0.03, *p* = 0.02) and less likely to be depressed than non‐native or dormitory students (β = −0.02, *p* = 0.02). The indirect effect of prayer on depression was also significant. This means that prayer was associated with a decrease in depression (β = −0.131, *p* = 0.03), but this effect was mediated by healthy behaviors, especially non‐smoking.

In addition to statistical significance, we examined the practical relevance of our findings by interpreting effect sizes (standardized coefficients, β). According to commonly cited benchmarks [33], β values of ± 0.10, ± 0.30, and ± 0.50 represent small, medium, and large effects, respectively. In our model, the direct association between prayer and non‐smoking (β = 0.27) approached a medium effect, while the indirect association between prayer and depression (β = −0.13) was small. These effect sizes suggest that although some associations reached statistical significance, their practical relevance may be modest and should be interpreted cautiously.

In other words, prayer is associated with an increased likelihood of non‐smoking, which in turn is associated with a decreased risk of depression. The results of this study suggest that prayer, native‐ness, and non‐smoking are all important factors in the prevention of depression among students, and non‐smoking was the mediator variable in the association between prayer and depression in students.

## 7 Discussion

Prayer is a fundamental act of worship for Muslims and can positively impact physical and mental health. The current study demonstrated a significant direct relationship between commitment to prayer and healthy eating behavior, indicating that increased frequency of prayer is associated with greater adherence to healthy eating principles. Previous research has shown a direct correlation between prayer and the consumption of fruits and vegetables [34, 35]. Additionally, parental religiosity influences their children's healthy eating behaviors [36]. Nurmansyah's study revealed a positive and significant relationship between prayer and healthy eating behavior, with those who pray at least five times a day adhering more to healthy eating principles [37]. These studies suggest that prayer, as a spiritual activity, can positively influence healthy eating behavior. However, given the cross‐sectional design of the present study, these findings reflect associative relationships rather than causal effects, and interpretations should remain cautious.

The results also showed an inverse direct relationship between commitment to prayer and smoking; prayer is associated with an increased likelihood of not using tobacco, which in turn is linked to a reduced risk of depression. The more one is prayer, the less one tends to smoke. Abdoljabari's study indicated that smoking among students who pray is significantly less than among those who do not [38]. Furthermore, research by Bai and Purwono showed a significant difference between prayer and preventive behaviors against smoking and alcohol consumption, with those praying at least five times a day having lower rates of smoking and alcohol use. These studies indicate that prayer, as a spiritual activity, can positively impact behaviors that prevent smoking and alcohol consumption [39, 40]. In the present study, the indirect effect of prayer on depression through smoking behavior was statistically significant; however, this protective pathway should be interpreted cautiously due to the indirect and limited nature of the observed effects. Therefore, the role of prayer is described as an associated factor rather than a definitive protective determinant.

Al‐Kandari and Najam's studies have shown that prayer can reduce nervous tension and help reduce stress and anxiety [23, 24]. While these findings support a potential beneficial association, the current study does not establish a causal mechanism, and further longitudinal research is required to confirm these relationships. Prayer may contribute to reduced depression indirectly by promoting healthier behaviors such as reduced smoking. Leung and colleagues stated that praying increases positive religious emotions such as love, gratitude, hope, and peace, and these emotions may reduce depressive symptoms [41].

In their research, Jakubowska et al showed that regular physical activity effectively reduces depression symptoms and increases cognitive functions and acts as a useful complementary treatment for depression management [42]. The present study also showed that physical activity had a positive effect on reducing depression symptoms, which is consistent with the mentioned study on the other hand, physical activity positively effects reducing depression symptoms. Also, Hu and Jacinto showed that regular exercise helped improve mood and could be used as a complementary treatment for depression. Exercise releases endorphins in the brain, known as happiness hormones, which can enhance individual well‐being [43, 44]. The current study also showed that physical activity among female students is higher compared to male students. This finding should be interpreted in light of cultural and contextual characteristics of the study population, and differences with some international studies may reflect sociocultural factors, access to facilities, and lifestyle patterns.

Studies show that there is a strong correlation between prayer observance and improved student learning outcomes. Students who engage in prayer regularly tend to achieve better academic results [45, 46] However, although previous studies have not consistently reported a decline in prayer commitment across academic terms, the present study found that commitment to prayer decreased with increasing semester level. This may be related to academic workload, stress, changing priorities, and environmental factors. Importantly, given the observational nature of the study, these associations do not imply that academic progression directly causes reduced religious commitment; rather, they indicate a statistical relationship that warrants further investigation.

Encouraging students to pray is directly related to their participation in congregational prayers. The current study found that adherence to prayer decreased with increasing academic terms. This finding should be interpreted cautiously and may reflect contextual influences rather than a deterministic trend. Local students scored higher in prayer adherence than non‐local or dormitory students. Kavosi showed that local students had greater practical commitment to prayer than non‐local students [1]. These differences likely reflect cultural, familial, and environmental support systems, highlighting the importance of contextual factors in shaping religious behaviors.

Ghodratnama's study showed a significant positive relationship between socio‐economic status and physical activity [47], which contrasts with the current findings. Such inconsistencies may be attributed to contextual, lifestyle, and environmental differences between populations, emphasizing the need for cautious comparison with international literature.

Although we observed non‐significant associations between prayer and some health behaviors (physical activity and certain dietary indices) in our sample, this finding is consistent with the broader literature that reports mixed or context‐dependent relationships between religiosity/spirituality and health behaviors. Systematic reviews and population studies have shown positive associations with fruit/vegetable intake but mixed results for fat intake and other dietary measures [48], and several large community studies have found no clear association between spirituality and levels of physical activity while showing effects on related behaviors such as sedentary time [49]. Moreover, recent systematic reviews conclude that evidence linking religiosity/spirituality to physical activity is heterogeneous and not yet robust enough to support causal claims, largely due to cross‐sectional designs, varied religiosity/spirituality measures (including single‐item or private‐devotion indicators), and population/cultural differences across studies [50, 51]. Accordingly, the non‐significant associations were interpreted cautiously, and possible explanations were discussed in terms of methodological characteristics of the study, measurement limitations, and contextual factors. These findings highlight the need for future longitudinal and methodologically robust research to better clarify the relationships between prayer and health behaviors.

## 8 Limitations

One limitation of this study was the absence of students attending the faculty and internships, which was solved by coordinating with the internship unit or inviting students to the faculty. Given the cross‐sectional design of the study, causal inferences cannot be made, and the observed relationships should be interpreted as associations rather than cause–effect relationships. This represents a fundamental methodological limitation.

Another limitation of the current study was the fear of the correct answer, which was done with the following measures to adjust this problem: The questionnaires were designed anonymously and the names and surnames of the students were not included in them. Also, the interviewers were not selected from university professors. Before the beginning of the questioning process, the purpose of the study was fully explained to the students and it was emphasized that the collected information will remain completely confidential and will not have any effect on the grades and courses of the students.

Despite these precautions, the possibility of social desirability bias and religious response bias cannot be completely ruled out due to the self‐report nature of the instruments.

Furthermore, the generalizability of the findings is limited because the sample was drawn from a single medical sciences faculty. Therefore, caution should be exercised when extending the results to other universities or populations.

Several measurement limitations should be acknowledged, including the use of single‐indicator constructs, dichotomous response formats for some items, and marginal convergent validity (AVE) for certain subscales. We did not test alternative competing models (e.g., direct‐only or different mediation pathways), nor did we examine whether the mediation model differs across demographic subgroups using multi‐group analysis, as moderation was not a primary aim. Future research should address these limitations by using more refined measures, testing multiple competing models, and exploring moderated mediation in larger samples.

## 9 Conclusion

The results of this study show that adherence to prayer as a spiritual act was associated with positive effects on healthy behaviors and mental health among students. However, given the cross‐sectional design of the study, these findings reflect statistical associations rather than causal relationships. Therefore, conclusions should be interpreted cautiously and within the limits of the data.

By increasing students' awareness and positive attitude towards prayer, healthy behaviors may potentially be promoted among them. Nevertheless, any policy or intervention recommendations should be considered preliminary and require further empirical confirmation.

In addition, it is necessary to develop educational and cultural programs to encourage students to pray and emphasize its positive effects on physical and mental health. Such recommendations should be viewed as context‐based suggestions rather than definitive evidence‐based interventions, given the descriptive nature of the present study.

Future research is recommended to employ longitudinal, multi‐center, and experimental (interventional) designs to more rigorously examine the relationships between adherence to prayer, healthy behaviors, and depression. Using more diverse methodological approaches and validated measurement tools in different populations will help clarify causal pathways and strengthen the evidence base in this field.

## Author Contributions

**Mehrdad Karimi:** conceptualization, investigation, funding acquisition, writing – original draft, formal analysis, software. **MoradAli Zareipour:** conceptualization, investigation, funding acquisition, writing – original draft, writing – review and editing, visualization, project administration, resources, supervision. **Esmayil Zeynali:** data curation, writing – original draft, funding acquisition. **Rahim Sharafkhani:** methodology, validation.

## Ethics Statement

This research project was conducted by the Ethics Committee of Khoy University of Medical Sciences with the ethical code IR.KHOY.REC.1402.006. All ethical considerations, including confidentiality of information, anonymity of questionnaires, were carefully observed. And ensuring the consent of parents and students to participate in the study. The university's ethics committee emphasized the importance of informed consent from students and parents. During the data collection process involving students, written informed consent was obtained from both the students and their parents.

## Conflicts of Interest

The authors declare no conflicts of interest.

## Transparency Statement

Dr. Morad Ali Zareipour, author, affirms that this manuscript is an honest, accurate, and transparent account of the study being reported; that no important aspects of the study have been omitted; and that any discrepancies from the study as planned (and, if relevant, registered) have been explained.

## Data Availability

The data that support the findings of this study are available on request from the corresponding author. The data are not publicly available due to privacy or ethical restrictions.

## Footnotes

1 Degree of Freedom.
